# Multi-walled carbon nanotubes elicit concordant changes in DNA methylation and gene expression following long-term pulmonary exposure in mice

**DOI:** 10.1016/j.carbon.2021.03.045

**Published:** 2021-06

**Authors:** Giovanni Scala, Mathilde N. Delaval, Sourav P. Mukherjee, Antonio Federico, Timur O. Khaliullin, Naveena Yanamala, Liliya M. Fatkhutdinova, Elena R. Kisin, Dario Greco, Bengt Fadeel, Anna A. Shvedova

**Affiliations:** a Department of Biology, University of Naples, Naples, Italy; b Institute of Environmental Medicine, Karolinska Institutet, Stockholm, Sweden; c Faculty of Medicine and Health Technology, Tampere University, Tampere, Finland; d Health Effects Laboratory Division, NIOSH, CDC, Morgantown, WV, USA; e Department of Hygiene and Occupational Medicine, Kazan State Medical University, Kazan, Russia; f Department of Physiology and Pharmacology, West Virginia University, Morgantown, WV, USA

**Keywords:** Carbon nanotubes, DNA methylation, Epigenetics, Inflammation, Transcriptomics

## Abstract

Pulmonary exposure to multi-walled carbon nanotubes (MWCNTs) causes inflammation and fibrosis. Our previous work has shown that industrially produced MWCNTs trigger specific changes in gene expression in the lungs of exposed animals. To elucidate whether epigenetic effects play a role for these gene expression changes, we performed whole genome bisulphite sequencing to assess DNA methylation patterns in the lungs 56 days after exposure to MWCNTs. Lung tissues were also evaluated with respect to histopathological changes and cytokine profiling of bronchoalveolar lavage (BAL) fluid was conducted using a multi-plex array. Integrated analysis of transcriptomics data and DNA methylation data revealed concordant changes in gene expression. Functional analysis showed that the muscle contraction, immune system/inflammation, and extracellular matrix pathways were the most affected pathways. Taken together, the present study revealed that MWCNTs exert epigenetic effects in the lungs of exposed animals, potentially driving the subsequent gene expression changes.

## 1. Introduction

Pulmonary exposure to multi-walled carbon nanotubes (MWCNTs) *via* pharyngeal aspiration or inhalation has been shown to cause inflammation and fibrosis as well as systemic responses in a variety of animal models [1 ]. Furthermore, instillation of MWCNTs into the pleural cavity of mice was shown to trigger mesothelioma suggesting that certain long and fiber-like MWCNTs present the same carcinogenic hazard as asbestos (when injected into the pleura) [2].

Importantly, we and others have reported the occurrence of biomarkers of inflammation and fibrosis in workers manufacturing MWCNTs [3,4]. Epigenetic alterations were also documented in workers exposed to MWCNTs [5]. Hence, while the analysis of global DNA methylation levels did not reveal significant differences, the analysis of gene-specific DNA methylation showed significant changes in MWCNT-exposed workers compared to controls [5]. Epigenetic regulation of gene expression plays a critical role in the regulation of cellular processes. Epigenetic mechanisms include, for instance, DNA methylation, histone modification, chromatin remodeling, and small and long noncoding RNA (ncRNA) expression [6]. Several recent studies have disclosed changes in DNA methylation in human cell lines exposed *in vitro* to MWCNTs [7–9]. Evidence of DNA methylation changes corresponding to pulmonary inflammation were also reported in mice exposed acutely (up to one week) to MWCNTs [10,11 ]. However, there are few if any studies of genome-wide DNA methylation changes following *in vivo* exposure to MWCNTs. Moreover, in order to understand the role of these epigenetic changes for the toxicity of MWCNTs, the link between DNA hyper- or hypomethylation and the subsequent changes in gene expression also needs to be demonstrated [6].

Several *in vitro* [12,13] and *in vivo* studies [14,15] have addressed the toxicity of the “benchmark” MWCNT, Mitsui-7/MWCNT-7, a material classified by the International Agency for Research on Cancer (IARC) as being potentially carcinogenic to humans (Group 2B) [16]. However, as recently pointed out, studies of other, as-manufactured (non-purified) MWCNTs that may realistically affect workers are limited [17]. We previously reported the mRNA and ncRNA expression profiles in the blood of workers exposed to MWCNTs [18]. Using MWCNTs from the same manufacturing plant, we also conducted a comparative analysis of the lung and blood transcriptomes of mice [19]. Here, we addressed the question whether DNA methylation changes occur following pulmonary exposure to MWCNTs in mice and, furthermore, whether such epigenetic changes correlate with the gene expression changes in mice exposed to the same MWCNTs [18]. To this end, MWCNTs were administered to mice *via* pharyngeal aspiration and samples were collected for analysis 56 days post-exposure. We then performed an integrated analysis of DNA methylation and transcriptomics data in the lungs.

## 2. Experimental

### 2.1. Nanomaterial

MWCNTs were produced by NanoTechCenter, Ltd. (Tambov, Russia) by catalytic vapor deposition. MWCNTs had an outer diameter of 8–15 nm, inner diameter of 4–8 nm, and length of 2–15 μm. The total amount of metal impurities did not exceed 5%, the specific geometrical surface was 300–320 m^2^/g [19]. Endotoxin levels in all samples were below detection limit as assessed with the LAL chromogenic endpoint assay (Hycult Biotech, Inc., Plymouth Meeting, PA).

### 2.2. Animal model

Specific pathogen-free adult female C57BL/6 mice (7–8 weeks) were provided by Jackson Laboratories (Bar Harbor, ME). Animals were housed individually, receiving filtered high efficiency particulate air (HEPA) in the AAALAC International-accredited animal facility at NIOSH. All procedures complied with the ethical standards set forth by the Animal Welfare Act (enforced by the United States Department of Agriculture) and the Office of Laboratory Animal Welfare (OLAW). The studies were approved by the NIOSH Health Effects Laboratory Division (HELD) Institutional Animal Care and Use Committee within the Center for Disease Control (Public Health Services Assurance Number A4367–01) in accordance with an approved protocol.

### 2.3. Animal exposure

MWCNTs were administered to mice *via* pharyngeal aspiration, as previously described [20]. Briefly, after anesthesia with a mixture of ketamine (Phoenix, St. Joseph, MO) and xylazine (Phoenix, St. Joseph, MO) (62.5 and 2.5 mg/kg subcutaneous in the abdominal area), the mouse was placed on a board in a near vertical position and the animal’s tongue was extended with lined forceps. MWCNT suspensions (40 or 80 μg/mouse) were placed posterior in the throat and the tongue held until the suspension was aspirated. All animals from the control and MWCNT-exposed groups survived this procedure and exhibited no overt behavioral or health outcomes.

### 2.4. Histopathology

Lung tissues were harvested, and inflation fixed *in situ* with 4% paraformaldehyde at constant pressure of 10 cm H_2_O for 10 min with the chest cavity open. Coronal sections were cut from the lungs, embedded in paraffin, and sectioned at a thickness of 5 μm with an HM 320 rotary microtome (Carl Zeiss, Thornwood, NY). Sections were then stained with Masson trichrome, and histological evaluation was performed. Sample identification was coded to ensure unbiased evaluation.

### 2.5. BAL collection

Mice were weighed and sacrificed 56 days post-MWCNT exposure with an intraperitoneal injection of sodium pentobarbital (>100 mg/kg) and exsanguinated. The trachea was cannulated with a blunted 22-gauge needle, and bronchoalveolar lavage (BAL) was performed using cold sterile PBS [21]. Cell-free BAL aliquots were frozen at −80 °C until further analysis.

### 2.6. Cytokine analysis

Cytokines in BAL were analyzed using a Bio-Plex^®^ Pro Mouse Cytokine 23-plex assay (Bio-Rad, Hercules, CA). The concentrations were calculated using Bio-Plex^®^ Manager 6.1 software (Bio-Rad) based on standard curves. Results are presented as pg/mL based on results from 5 animals *per* exposure group. Data retrieved from the multiplex assay were analyzed using hierarchical clustering after normalization to the control group (log2 fold change), as described [22].

### 2.7. DNA extraction

Lung tissue samples from control and MWCNT-exposed mice were cut into small pieces (10–15 mg), snap frozen, and stored at −80 °C until further analysis. The frozen samples were thawed on ice and briefly homogenized in 200 μL PBS using a sterile Axygen^™^ Tissue Grinder (Fisher Scientific). The samples were then centrifuged at 1500 rpm for 15 min at 4 °C and supernatants were discarded. Genomic DNA was extracted from the cell pellet using QIAamp^®^ DNA Mini Kit (Qiagen, Sweden) following the manufacturer’s instruction. Briefly, 20 μL proteinase-K and 180 μL ATL buffer provided with the kit were added and mixed by vortexing, followed by incubation at 56 °C for 2 h to ensure complete lysis of the cells. The samples were vortexed occasionally during this incubation step. Then, 200 μL AL buffer was added and mixed thoroughly by vortexing for 15 s. The samples were then incubated at 70 °C for 10 min, followed by brief centrifugation to collect the droplets accumulated in the lids of the tubes. The samples were then mixed with 200 μL ethanol, and thereafter briefly centrifuged and transferred onto the Qiagen QIAamp Mini spin column and genomic DNA (gDNA) was isolated from the samples in 200 μL distilled water (RNase- and DNase-free). The gDNA concentrations were determined on a ThermoFisher NanoDrop instrument prior to sequencing.

### 2.8. Whole genome sequencing

The quality of the gDNA samples was first determined and the concentrations were calculated using Qubit/Quant-iT assay. The sequencing libraries of the gDNA samples were prepared using the TruSeq DNA Methylation kit (Illumina Inc.) according to the manufacturer’s protocol, followed by cluster generation and 150 cycle pair-end sequencing in 16 lanes using the HiSeqX system and v2.5 sequencing chemistry (Illumina Inc.). The sequencing depth was 400 million read pairs *per* lane, with at least 75% of the bases having a quality score of 30 or higher. Sequencing data were deposited in Zenodo (http://doi.org/10.5281/zenodo.4315419). Zenodo is hosted by CERN and is compliant with OpenAIRE (open access) guidelines version 3.0.

### 2.9. Methylation data preprocessing

The fastq-files were analyzed with the NGI MethylSeq pipeline: https://github.com/nf-core/methylseq/blob/master/docs/output.md. In particular, the following processing and analysis steps were executed. FastQC was used to control read quality before and after the removal of adapter contamination and trimming of low quality regions using TrimGalore (http://www.bioinformatics.babraham.ac.uk/projects/trim_galore/) with default parameters. Reads were aligned to the mm10 reference genome with Bismark [23]. Deduplication of clonal reads was performed with *deduplicate_bismark* script from the Bismark suite. Finally, cytosine methylation was called using the *bismark_methylation_extractor* module from the Bismark suite.

### 2.10. Differential methylation analysis

Samples were first filtered for CpG sites having at least 10x coverage. CpG methylation values were then considered over 100 bp bins using the *regionCounts* methylkit function [24] and regions covered in all samples were retained for testing for differential methylation. Differentially methylated regions were computed using the *calculateDiffMeth* function from the methylkit package with default parameters. To perform differential promoter methylation analysis, the list of mm10 protein-coding genes was obtained from UCSC “refseqCurated” tables and filtered for genes having a length greater than 2 kb. For each of the above defined genes, methylation calls were mapped to a region spanning from TSS-1000 to TSS+200 using the *regionCounts* function from the methylkit package. Only promoters present in all the samples were retained for subsequent analyses. Finally, differentially methylated regions were computed by applying the *calculateDiffMeth* function from the methylkit package using default parameters.

### 2.11. Integrative data analysis

Transcriptomics (mRNA) data were retrieved from Khaliullin et al. [19] (GSE140676). Specifically, lung tissue gene expression profiles of mice exposed to MWCNTs and control samples were downloaded, and differential expression analysis was performed between treated and control groups using the limma package. Differential methylation and differential transcriptional data were integrated using the SMITE toolkit [25]. The analyzed signals were integrated using mm10 protein-coding genes from the UCSC mm10 *ncbiRefSeqCurated* tables. Using the function *annotateExpression,* p-values and fold-change values were mapped from the differential gene expression analysis to the corresponding mm10 genes using the transcript with the lowest p-value in case of multiple transcripts mapping to the same gene. To map methylation values over gene promoters, the promoter region of each gene in question was defined as the region [TSS - 1 kb, TSS + 200 b] flanking its transcription start site using the *makePvalueAnnotation* function. Each promoter was associated with a set of overlapping regions from the differential methylation analysis using their genomic position by using the *annotateModification* function. For each promoter region, the associated region fold-change values and weighted p-values from the differential methylation analysis were weighted by the distance of the region from the TSS and summarized using the Stouffer method. Methylation p-values were then logit transformed and rescaled using the expression p-value distribution as reference using the *normalizePval* function. The function *scorePval* was then employed to assign a score to each gene, by integrating the expression p-value and fold-change with the corresponding value from promoter methylation using a model, defined with the *makePvalueObject* function, wherein promoter methylation is inversely correlated with gene expression.

Finally, to select relevant genes from this analysis, a filtering step was performed, retaining only those genes showing significant promoter methylation and transcription changes, and respecting one of the following patterns of concordance: promoter hypermethylation/gene downregulation, and promoter hypomethylation/gene upregulation. Venn diagrams were plotted with the Draw Venn Diagram tool (http://bioinformatics.psb.ugent.be/webtools/Venn/).

### 2.12. Functional analysis

The list of the selected concordant genes (*i.e.,* 100 top-scoring promoter hypermethylated and 100 top-scoring promoter hypomethylated, along with the associated transcription log-fold change values) were analyzed with the FunMappOne tool [26]. FunMappOne is a graphical tool developed by us that can be applied to display the enriched terms from the REACTOME database. For the enrichment analysis using FunMappOne, p-values < 0.05 were considered significant.

### 2.13. Statistical analysis

Cytokine results were analyzed by one-way ANOVA using Tukey’s multiple comparison test. Results are presented as mean values ± SEM and p-values < 0.05 were considered significant.

## 3. Results

### 3.1. MWCNT-induced lung pathology and cytokine responses

**Fig. 1.**
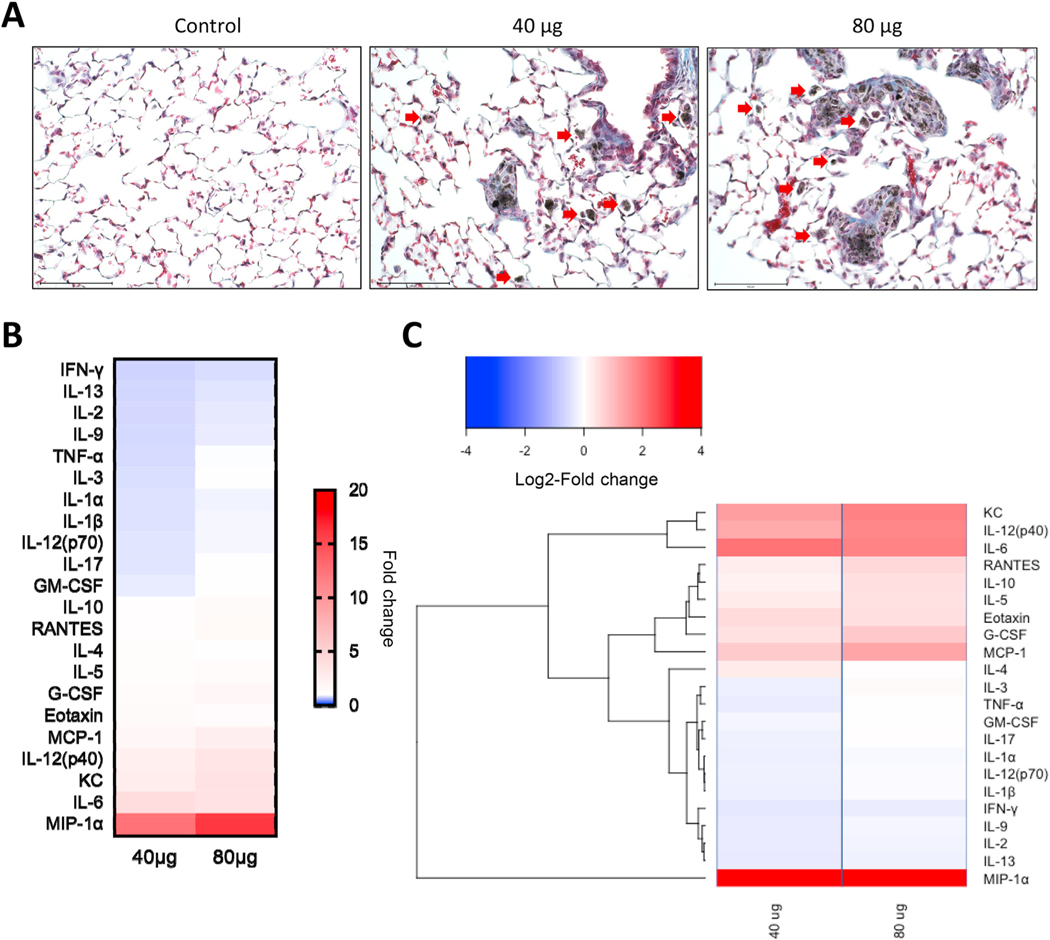
MWCNTs cause persistent lung damage and inflammation in mice. (A) Representative photomicrographs of lung tissues of C57BL/6 mice administered PBS (control), 40 μg, or 80 μg of MWCNTs *per* mouse. Granulomatous lesions and collagen deposition are noted in MWCNT-exposed animals. Arrows point to MWCNT-laden macrophages. (B) Cytokine profiling of mice exposed to MWCNTs (0, 40 and 80 μg) was performed at 56 days post-exposure using a multi-plex array. The color scale in the heatmap represents fold change values compared to control. (C) Hierarchical clustering analysis of the cytokine data (refer to Suppl. Fig. S1 for the quantitative data). The color scale represents the log2-fold change values.

C57BL/6 mice were exposed by pharyngeal aspiration to MWCNT suspensions (40 or 80 μg/mouse). At 56 days post-exposure, histological evaluation of lung tissue sections revealed granulomatous lesions, presence of individual and agglomerated macrophages with phagocytized MWCNTs (evidenced as dark particulate matter), and collagen deposition in all MWCNT-exposed mice, and the outcomes were aggravated at the highest dose compared to control (Fig. 1A). These results are consistent with the previous observations of Khaliullin et al. [19].

To identify the potential inflammatory effects of MWCNT at 56 days post-exposure, we analyzed the expression of 23 cytokines and chemokines in BAL samples from mice exposed to 40 and 80 μg of MWCNTs (Fig. 1B–C, and Suppl. Fig. S1). MWCNT exposure induced statistically significant changes in the level of expression of several inflammatory mediators in comparison to the control group. The levels of all measured chemokines (Eotaxin, KC, MCP-1, MIP-1α, and RANTES) were significantly elevated in a dose-dependent manner. Hierarchical cluster analysis (Fig. 1C), presented as log2-fold change values over control, showed a clear clustering of cytokines as indicated by the dendrogram to the left of the heatmap. Additionally, several cytokines including IL-1β and TNF-α were slightly albeit significantly reduced in BAL of mice exposed to 40 μg MWCNTs, but not in the high dose group.

### 3.2. MWCNTs trigger DNA methylation changes in mouse lungs

**Fig. 2.**
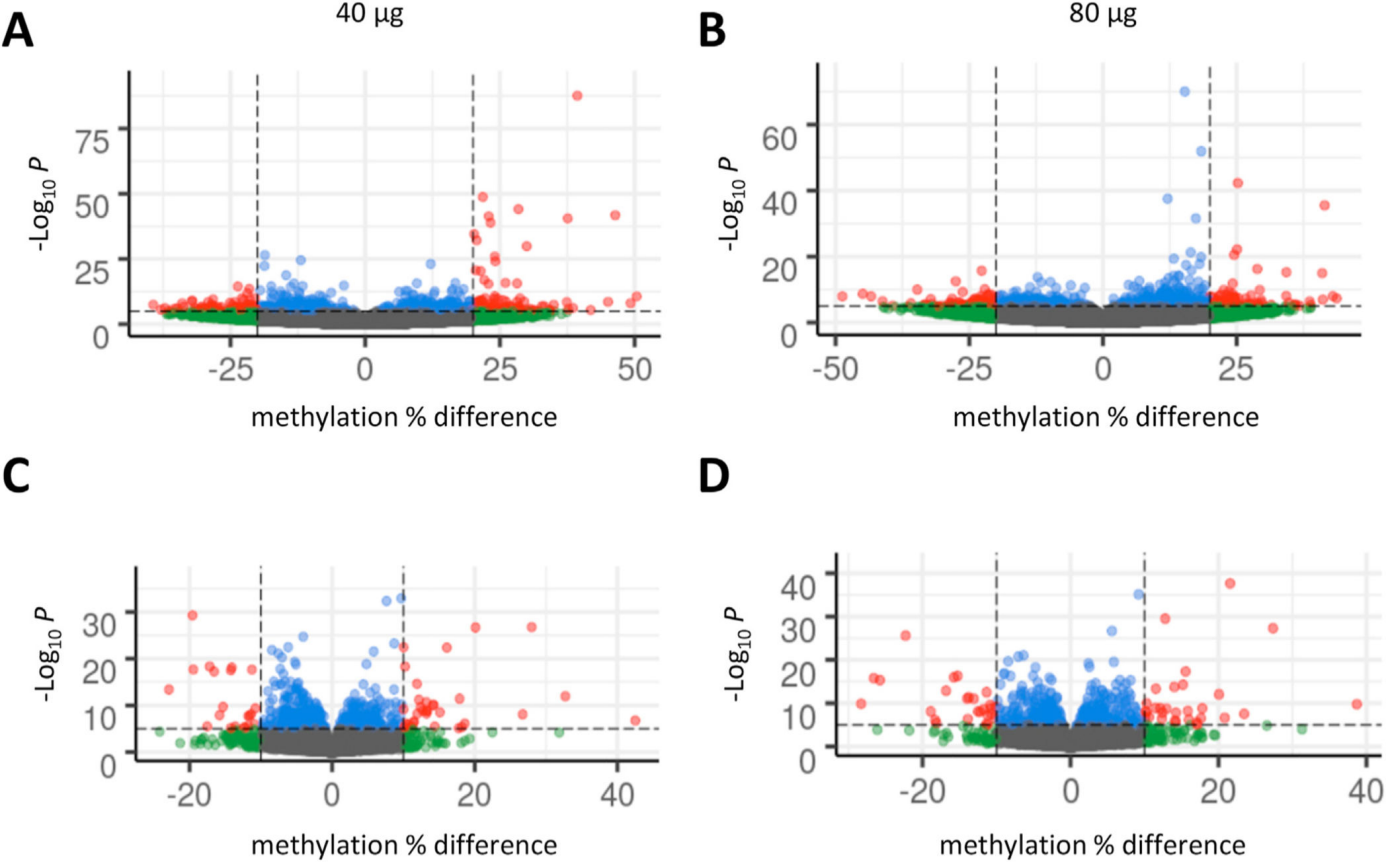
MWCNTs elicit DNA methylation changes in mouse lungs. DNA methylation analysis was performed at 56 days post-exposure in mice exposed to MWCNTs (40 or 80 μg *per* animal). Volcano plots depict the results of differential region or promoter methylation analysis for the 40 μg dose (A and C) and the 80 μg dose (B and D). The values on the x-axis report the observed differences in DNA methylation while values on the y-axis correspond to the -log10 p-values. Red circles represent regions with p < 10^−5^ and absolute methylation differences >20% in A and B and >10% in C and D, respectively, while blue circles represent regions with p < 10^−5^ and absolute methylation differences <20% in A and B and <10% in C and D; green circles represent regions with p > 10^−5^ and absolute methylation differences >20%.

We then investigated DNA methylation profiles using whole genome bisulfite sequencing. No appreciable changes linked to exposure were noted when looking at global methylation levels (Fig. S2). We therefore explored differentially methylated regions (DMRs), considering segments of 100 bp length across the entire genome and found 141 hypomethylated regions and 156 hypermethylated regions associated with the 40 μg exposure group and 153 hypomethylated regions and 139 hypermethylated regions associated with the 80 μg exposure group. The volcano plots in Fig. 2A and B shows the global trend in observed methylation differences for the 40 μg and 80 μg groups, respectively. The list of differentially methylated regions (qvalue < 0.05) for each group is reported in Suppl. Table S1. This analysis thus revealed a global pattern of hypermethylation linked to genomic DMRs at both doses (40 μg and 80 μg *per* mouse) compared to control samples. Next, in order to identify epigenetic aberrations having a potential transcriptional effect, we performed differential promoter methylation analysis [27]. For each gene, we considered its promoter region as comprised between 1000 bp upstream of the transcription start site (TSS-1000 bp) and 500 bp downstream of the transcription start site (TSS+500 bp) and examined the CpG methylation levels across these regions. We then explored differentially methylated promoters by comparing the methylation levels observed in control samples against the corresponding values observed in the 40 and 80 μg groups. The volcano plots in Fig. 2C and D shows the global trend in observed promoter methylation differences for the 40 μg and 80 μg groups, respectively. The corresponding list of differentially methylated promoters for each tested dose is reported in Suppl. Table S2. In total, we observed 136 significantly differentially methylated promoters in the 40 μg group (71 hypomethylated and 65 hypermethylated) and 115 significantly differentially methylated promoters in the 80 μg group (47 hypomethylated and 68 hypermethylated). Moreover, 39 promoters, out of a total of 212 promoters whose methylation levels were altered in at least one of the exposed groups, were found to be consistently altered in both 40 and 80 μg exposure groups. Genes involved in vesicle-mediated transport, such as *Trappc3l* (trafficking protein particle complex 3 like), were hypermethylated in both exposure groups.

### 3.3. Concordant changes in DNA methylation and gene expression

**Fig. 3.**
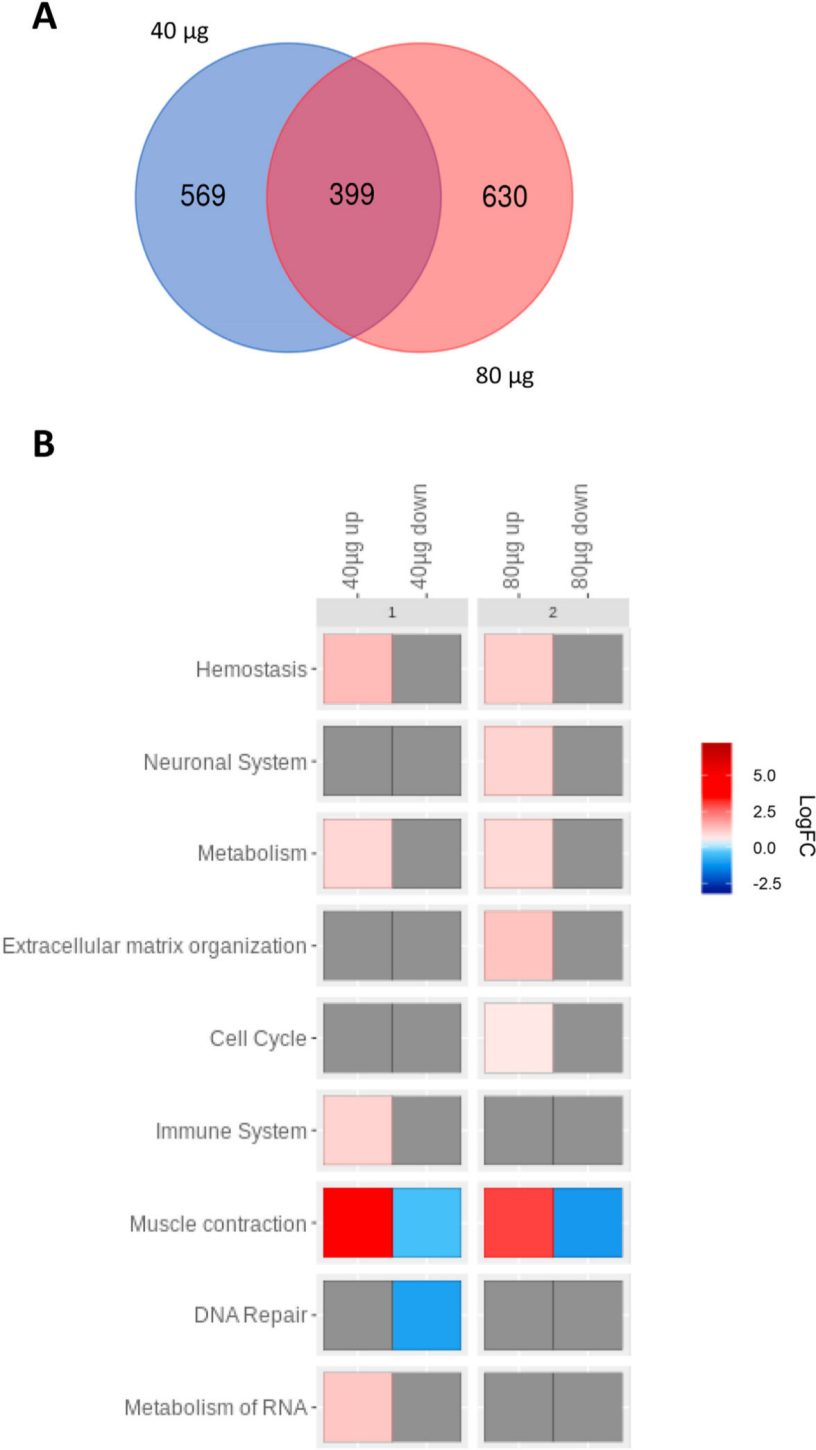
MWCNTs affect pathways related to immune signaling and muscle contraction. Pathway analysis was performed on the top-scoring concordant genes in the lungs of mice exposed to 40 or 80 μg MWCNTs at 56 days post-exposure. (A) Venn diagram representing significantly differentially expressed genes (DEGs) displaying concordant DNA methylation. (B) Functional analysis of the top-100 scoring genes was performed using the REACTOME database and visualized using FunMappOne. The color scale represents the average expression logFC.

In order to investigate potential transcriptional effects mediated by the observed changes in DNA methylation patterns after exposure to MWCNTs, we integrated our data with the previously obtained transcriptomics data from mice exposed to 40 μg of the same MWCNTs [19]. Thus, starting from the individual gene expression profiles, we first performed a differential expression analysis between exposed samples (*i.e.,* 40 μg of MWCNTs) and control samples and then integrated this differential gene expression information with the DNA methylation data for each tested dose. Table S3 reports the expression differences for each of the RefSeqCurated mouse genes integrated with DNA methylation changes observed in the 40 μg MWCNT and the 80 μg MWCNT samples. Our analysis of samples exposed to the 40 μg MCWNT dose identified a list of 968 genes showing significant changes both in their promoter methylation levels as well as in their transcription levels and respecting a pattern of promoter hypermethylation/gene downregulation or promoter hypomethylation/gene upregulation (denoted here as a concordant pattern of methylation/gene expression). The same analysis repeated by associating methylation differences in the 80 μg MWCNT exposure group revealed 1029 genes with significant epigenetic/transcriptional alterations and respecting the same pattern of concordance described above. Venn analysis showed a substantial overlap of the differentially expressed genes or DEGs between the 40 and 80 μg groups (Fig. 3A). Interestingly, among those genes in common between the 40 μg and 80 μg groups, *IL33* (interleukin-33), *ITIH4* (inter-alpha-trypsin inhibitor heavy chain H4), *TNFSF13* (tumor necrosis factor ligand superfamily member 13), and *TIMP1* (TIMP metallopeptidase inhibitor 1) showed the highest concordance scores. In addition, numerous cytokine- and inflammation-related genes with high concordance scores were found in 40 and 80 μg groups, including the IL-33 encoding gene which had the highest score (Table 1). Furthermore, *MYH1* (myosin heavy chain 1), was the second highest scoring gene with respect to concordance (in the 40 μg group) (Table S3).

**Fig. 4.**
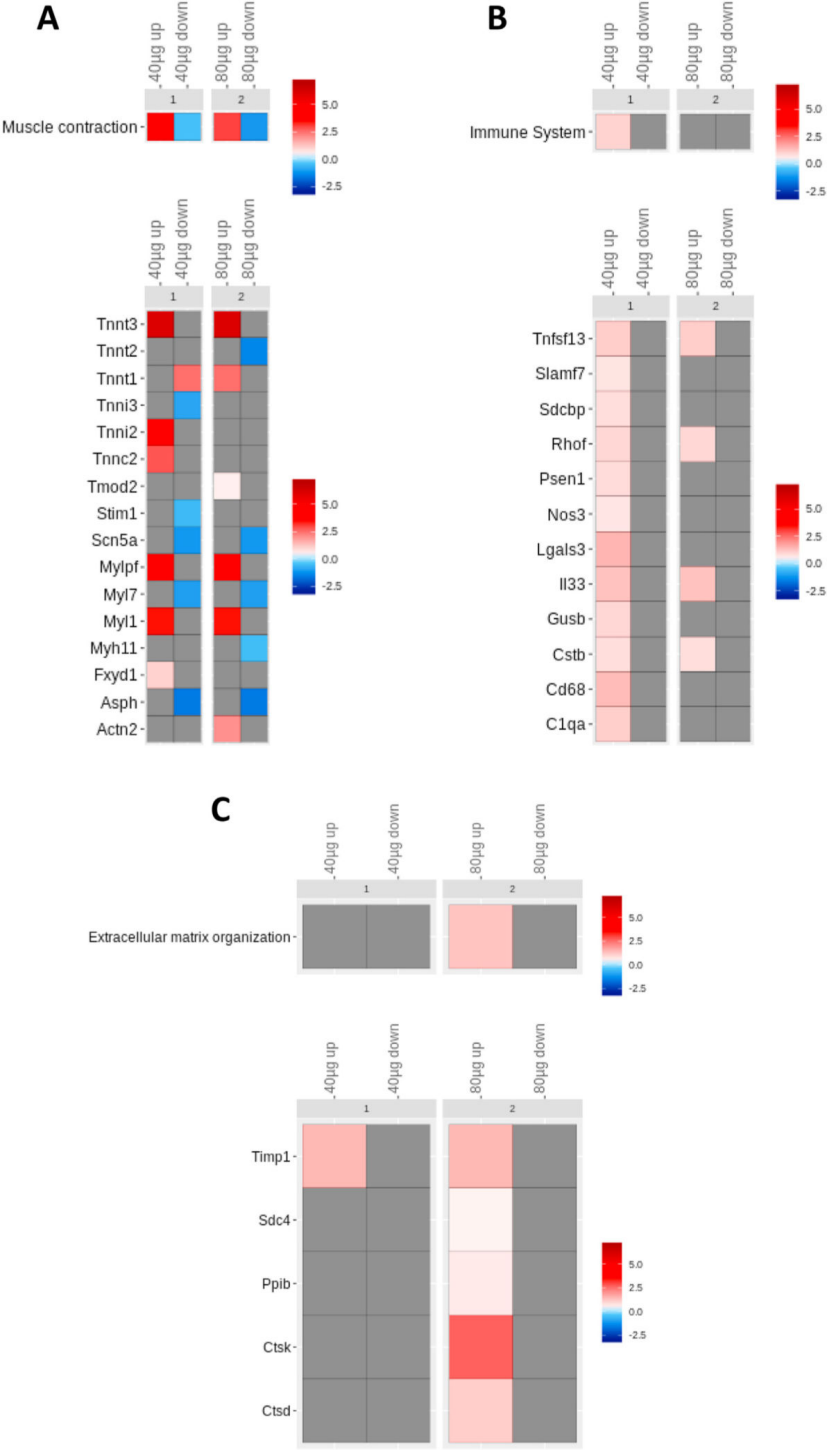
MWCNTs affect pathways related to muscle contraction, immune signaling and extracellular organization. The pathway enrichment analysis was performed on the top-scoring concordant genes identified in the integrative analysis. The up- and downregulated genes involved in the muscle contraction (A), immune system (B), and extracellular matrix pathways (C) are shown. The color scales represent the expression logFC of the genes in the pathways shown.

In order to investigate the functions that are dysregulated by genes whose alteration is potentially maintained by the observed epigenetic changes, we took the top-scoring concordant genes identified in the integrative analysis and performed a pathway enrichment analysis. Functional analysis using REACTOME allowed us to identify significantly altered pathways (Fig. 3B, Suppl. Tables S4–5). Notably, the muscle contraction pathway (Fig. 4A) was the most significantly altered pathway and it was dominated by upregulated genes in both 40 and 80 μg exposure groups. Several other pathways were dominated by upregulated genes, including the immune pathway for the low dose (40 μg) group (Fig. 4B), and the extracellular matrix organization pathway for the high dose (80 μg) group (Fig. 4C). The DNA repair pathway, however, was dominated by downregulated genes (Fig. 3B). The top-scoring genes, *IL33* and *TIMP1,* belonged to these significantly altered pathways (Tables S4–5). *MYH1* was also one of the top-scoring genes, as noted above. However, for unknown reasons, the REACTOME database does not annotate *MYH1* for *Mus musculus,* and therefore we could not ascribe *MYH1* to these pathways, even though the gene belongs to the muscle contraction pathway.

## 4. Discussion

MWCNT exposure through inhalation is the most likely scenario in the occupational setting [28]. Previous animal studies have shown that pulmonary damage/fibrosis caused by MWCNTs manifested at 28 days and progressed until 56 days [19]. For this reason, we chose to evaluate the effects on DNA methylation *versus* gene expression at 56 days post-exposure. The mice were exposed through pharyngeal aspiration, a method that corresponds well with inhalation exposure with respect to typical histopathological signs of inflammation and fibrosis [20], and with comparable biological responses as determined by gene expression analysis [29]. Here, granulomatous lesions and collagen deposition (fibrosis) were observed at day 56, and high dose MWCNTs (80 μg/mouse) triggered a stronger response compared to the low dose (40 μg). Furthermore, we found that MWCNTs elicited specific changes in DNA methylation at both doses. Based on our integrative analysis of DNA methylation and gene expression data, we provided evidence of about 1000 genes (968 genes in the 40 μg group, and 1029 genes in the 80 μg group) displaying significant and concordant changes in promoter methylation levels as well as their transcription levels. Functional analysis of the top-100 genes showed that immune-related pathways were among the most affected pathways, in line with the histopathological findings and the results of cytokine profiling performed on BAL fluid samples.

Interestingly, we identified *IL33* as the gene with the highest score with respect to concordant changes in gene promoter methylation and gene expression in mice exposed to MWCNTs for 56 days. IL-33, a member of the interleukin (1L)-1 cytokine superfamily, acts both as an extracellular cytokine that binds to ST2 (also known as 1L-1 receptor-like 1 or 1L1RL1) and as an intracellular factor that translocates into the nucleus, where it modulates gene expression [30]. Brown and co-workers have previously shown, using ST2-deficient [31] and 1L33-deficient mice [32], that the 1L-33-ST2 signaling axis is critically important for the orchestration of adverse pulmonary and extra-pulmonary responses upon exposure to MWCNTs. Our findings showed that 1L-33, sometimes referred to as a danger signal, is subject to epigenetic regulation in the lungs. The present findings were derived from homogenized lung tissue samples containing different cell types. Further studies are warranted to elucidate the cell type(s) implicated in epigenetic regulation of 1L-33 as well as the cellular targets of 1L-33. T_H_2 cells are central to the pathogenesis of many allergic inflammatory diseases [33] and may also play a role in the chronic pulmonary inflammation caused by carbon nanotubes [34–36].

The second-highest scoring gene with respect to concordance between DNA methylation and gene expression was *MYH1,* encoding a major contractile protein found in striated muscles. The role of the myosin-1 protein (also known as striated muscle myosin heavy chain 1) is to convert chemical energy into mechanical energy through the hydrolysis of ATP. The muscle contraction pathway featured prominently in our functional evaluation (even though *MYH1* was evidently not annotated in this pathway). Recent studies have shown that CNTs trigger the accumulation and activation of myofibroblasts in the lungs [37]. However, myofibroblasts display features of smooth muscle cells and fibroblasts, but not striated muscle cells, whereas in the present study, the muscle contraction pathway which is related to striated skeletal muscles was shown to be affected. On the other hand, the presence of striated muscle cells in non-neoplastic lung tissue may potentially arise through the myoblastic differentiation of mesenchymal cells. Moreover, it is notable that in addition to skeletal muscle stem cells (so-called satellite cells), several other cell types including pericytes, endothelial cells, and interstitial cells are known to display some myogenic potential [38]. Furthermore, it is relevant to note that single- and multi-walled CNTs have been shown to modulate mesenchymal stem cells [39,40]. 1n fact, Zhao et al. [41] reported that PEGylated MWCNTs directed myogenic differentiation of human mesenchymal stem cells in the absence of myogenic induction factors. Other investigators suggested that MWCNTs modulate myogenic differentiation of murine C2C12 myoblast cells through binding to bone morphogenetic protein receptor 2 (BMPR2) [42]. Taken together, it is possible to speculate that MWCNTs could act on (stem) cells with myogenic potential present in the lungs. However, further studies are needed to address this idea.

*Timp1* was also identified as one of genes showing the highest concordance between DNA methylation and gene expression changes. T1MP1 is secreted by activated macrophages and other cells to inhibit matrix metalloproteinases (MMPs) and thus plays a role in matrix remodeling. T1MP1 was recently shown to be strongly induced by MWCNTs [43], and the authors speculated that T1MP1 expression can be upregulated by activation of the transcription factor nuclear factor-kB (NF-kB). However, based on the present findings, epigenetic regulation of TIMP1 expression may be a more relevant (or alternative) mechanism. Using *Timpl* knockout mice, the authors could show that TIMP1 is a mediator of MWCNT-induced lung fibrosis. They found that myofibroblasts were induced in a TIMPl-dependent manner [43]. These findings also serve to underscore that non-immune cells, along with immune cells, are involved in adverse pulmonary effects including lung fibrosis and lung diseases like asthma [44].

Additionally, *ITIH4* showed one of the highest concordant scores in both the 40 μg and the 80 μg MWCNT samples. However, the latter gene was not involved in any of the most significantly altered pathways (*i.e*., muscle contraction, immune system, and extracellular matrix organization). Instead, it can be found in the hemostasis pathway (platelet activation, signaling and aggregation). ITIH4, inter-alpha-trypsin inhibitor heavy chain H4, is a protease inhibitor and is considered as an acute phase protein [45]. Interestingly, ITIH4 has been proposed as a biomarker of exposure to particulate matter ≤10 μm (PM_10_) [46]. Furthermore, *TNFSF13* is also among those genes with the highest concordance, and it encodes APRIL (“a proliferation-inducing ligand”), a member of the TNF ligand superfamily, that is thought to play a role in autoimmune disease [47] and suppresses allergic lung inflammation in mice [48].

There are some limitations to the present study including those related to the non-physiological pharyngeal aspiration approach yielding a bolus dose of MWCNTs. Additionally, as-produced MWCNT samples may contain metal impurities; however, as discussed above, the objective of the present study was to focus on as-produced MWCNTs that may realistically affect workers. Specifically, our samples contained <5% of metal impurities, mostly nickel and cobalt residues. Of those, nickel has been shown previously to increase the inflammogenic potential of MWCNTs using primary murine alveolar macrophages [49]. Furthermore, Aldieri et al. [50] reported, using the murine alveolar macrophage cell line MH-S, that iron-rich MWCNTs displayed cytotoxicity and genotoxicity, while iron-free MWCNTs did not exert adverse effects. Nevertheless, although metal impurities, especially transition metals, can contribute to the acute inflammogenic potential of MWCNTs, the morphology of MWCNTs is considered the major determinant of their toxicity [51]. Notwithstanding, the present study has taken into consideration the real-life human exposure scenario [3,4] by focusing on unpurified MWCNTs.

## 5. Conclusions

Pulmonary exposure to as-manufactured MWCNTs triggers inflammation and fibrosis in mice [19,21]. Using a genome-wide approach, the present study has revealed specific DNA methylation changes that correspond to changes in gene expression in mice 56 days post exposure to MWCNTs. Our results showed that immune-related pathways are among the most affected pathways while muscle contraction and extracellular matrix organization pathways were also perturbed following pulmonary exposure to MWCNTs. These results thus provide evidence of epigenetic regulation of gene expression in the lungs of mice exposed to MWCNTs. Further studies are needed to identify biomarkers of exposure for occupational surveillance [52]. The present results have implicated immune-related markers as promising candidates.

## Supplementary Material

supplemental

Table S1

Table S2

Table S3

## Figures and Tables

**Table 1:** Inflammation or immune-related genes among the concordant genes (*i.e.,* hypomethylation/upregulation, hypermethylation/downregulation) in the MWCNT (40 μg) group.

Gene	Name	Score	Rank	−log10(p-value)

*1133*	Interleukin 33	20,0637	1	4,6064
*Tnfsf13*	Tumor necrosis factor ligand superfamily member 13	16,34146	7	3,5200
*Mapkl4*	Mitogen-activated protein kinase 14	13,3944	30	3,7389
*Lta*	Lymphotoxin-alpha (LT-α) (or, TNF-β)	12,2235	53	2,6575
*Ccl3*	Chemokine (C–C motif) ligand 3 (or, MIP-1α)	10,6629	108	3,0807
*IH8rl*	interleukin-18 receptor 1	9,9544	156	1,6716
*Cd72*	Cluster of differentiation 72	9,5597	191	1,9861
*Il7r*	Interleukin-7 receptor	8,3746	340	2,7606
*Cd14*	Cluster of differentiation 14	7,9554	410	2,2024
*Csf1*	Colony stimulating factor 1 (CSF1) (or, M-CSF)	7,7815	447	1,7278
*Il10ra*	Interleukin-10 receptor subunit alpha	7,6511	474	1,4857
*Ccl21a*	C–C motif chemokine 21a	7,6248	481	2,2546
*Ilf2*	Interleukin enhancer-binding factor 2	7,4480	517	1,7869
*Ccr7*	C–C chemokine receptor type 7 (or, CD197)	6,9785	621	1,7711
*Cd5*	Cluster of differentiation 5	6,7982	664	1,7060
*Cxcl12*	C-X-C motif chemokine 12 (or, SDF1)	6,77883	670	1,6958
*Stat4*	Signal transducer and activator of transcription 4	6,6997	690	1,7085
*Cd160*	Cluster of differentiation 160	6,4776	735	1,5251
*Il2rb*	Interleukin-2 receptor subunit beta (or CD122)	6,1413	806	1,8947
*Tnfrsf9*	Tumor necrosis factor receptor superfamily member 9	6,0173	828	1,3272
*Cd93*	Cluster of differentiation 93	5,6067	903	1,6573
*Cd47*	Cluster of differentiation 47 (or, IAP)	5,3546	942	1,3875
*Il9r*	Interleukin-9 receptor (or, CD129)	5,2532	950	1,3916
