# Supplementary material for: Multi-walled carbon nanotubes elicit concordant changes in DNA methylation and gene expression following long-term pulmonary exposure in mice
Source: Carbon N Y. Author manuscript; Available in PMC 2023 May 18. (PMC10193301; doi:10.1016/j.carbon.2021.03.045)
Supplement: supplemental [file NIHMS1866907-supplement-supplemental.pdf]

Supporting Information

Multi-walled carbon nanotubes elicit concordant changes in DNA methylation and gene expression following long-term pulmonary exposure in mice

Giovanni Scala, Mathilde N. Delaval, Sourav P. Mukherjee, Antonio Federico, Timur O. Khaliullin, Naveena Yanamala, Liliya M. Fatkhutdinova, Elena R. Kisin, Dario Greco*, Bengt Fadeel*, and Anna A. Shvedova*

*Corresponding authors. E-mail: dario.greco@tuni.fi; bengt.fadeel@ki.se; ats1@cdc.gov

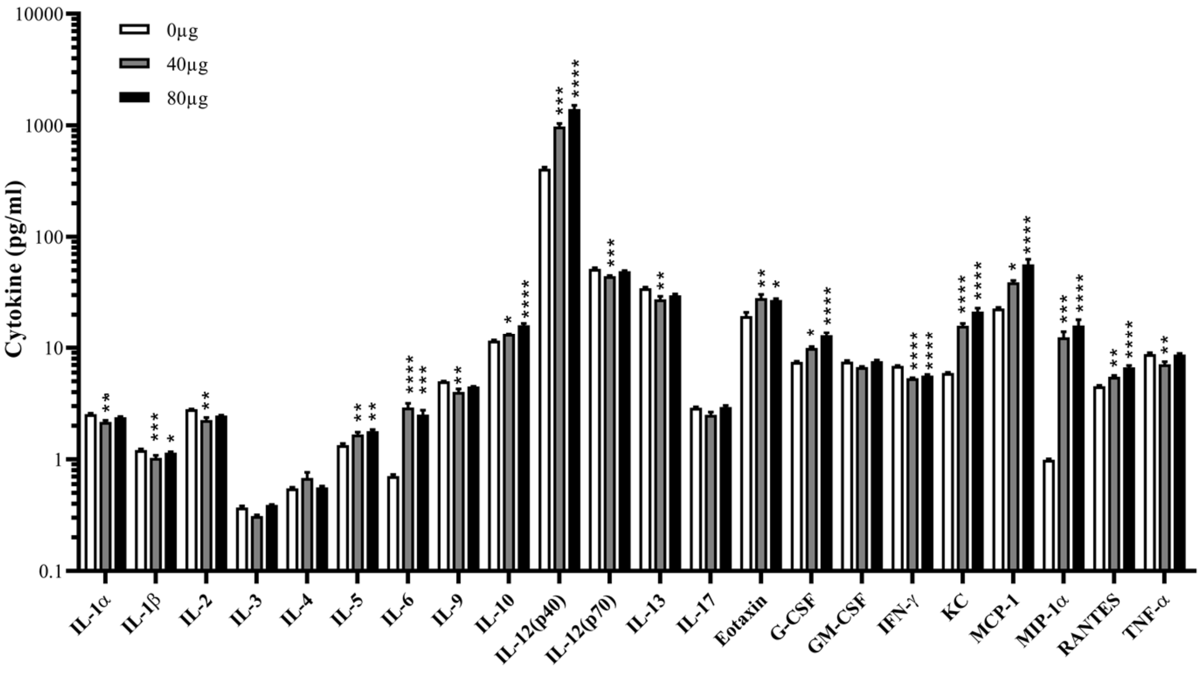

Figure S1. Cytokines/chemokines/growth factors in BAL fluid of mice exposed to MWCNTs (0, 40, 80 μg) analyzed at day 56 post-exposure using a 23-plex assay (cf. Figure 1).

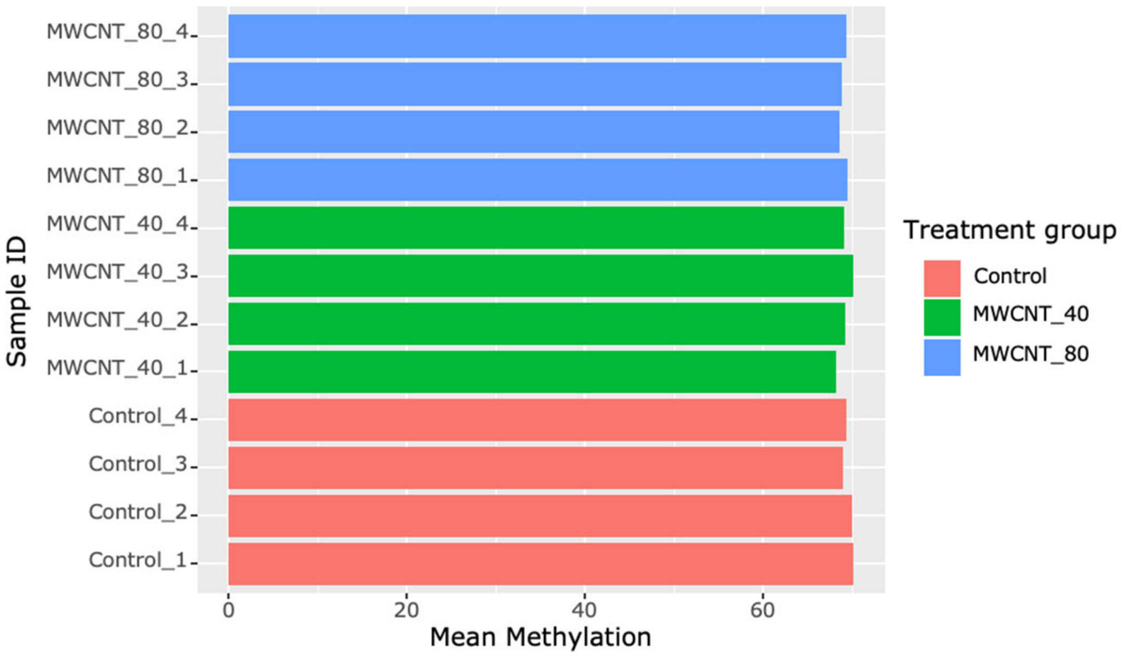

Figure S2. No changes in global methylation levels were observed. The average CpG methylation values in samples obtained at day 56 post-exposure from mice exposed to MWCNTs (0, 40 and 80 μg).

Table S4. REACTOME enriched pathways (hypomethylated / upregulated genes).

**Pathways**	**Group**	**P-value**	**Number of genes**	**Genes**
Muscle contraction	40µg	0,000185	6	TNNC2, MYLPF, TNNI2, FXYD1, TNNT3, MYL1
	80µg	0,000152	6	MYLPF, TMOD2, ACTN2, TNNT3, MYL1, TNNT1
Hemostasis	40µg		4	TIMP1, LHFPL2, ITIH4, IGF1
	80µg	0,000348		VAV1, TIMP1, SDC4, RAPGEF3, ITIH4, IRF2, GATA2, DAGLA, ACTN2
Metabolism	40µg	0,00732	13	CKB, NDUFA1, XYLT2, ARL2, ACOT9, GUSB, NOS3, PI4K2B, ADIPOR2, UROS, NDUFB11, B3GNT7, ACAD11
	80µg	0,0000378	16	CKB, AS3MT, B4GALNT1, SDC4, XYLT2, PSMB5, RAPGEF3, GPD1,ACOT9, GLB1L, BCAT2, UROS, SLC37A2, ATP5L, PI4KA, FITM2
Immune System	40µg	0,00455	13	CSTB, CD68, PSEN1, IL33, GUSB, SDCBP, NOS3, RHOF, C1QA, SLAMF7, LGALS3, MYH1, TNFSF13
Metabolism of RNA	40µg	0,00661	4	RPL3L, RPS25, RPL10A, RPL36A
Extracellular matrix	80µg	0,0458	5	TIMP1, CTSD, SDC4, CTSK, PPIB
Cell Cycle	80µg	0,0152	7	PSMB5,DYRK1A,UIMC1,POLE3,LCMT1,CDK6,RPS27
Neuronal System	80µg	0,0292	2	KCNN4, KCNMB4

Table S5. REACTOME enriched pathways (hypermethylated / downregulated genes).

**Pathways**	**Group**	**P-value**	**Number of genes**	**Genes**
Muscle contraction	40µg	0,00284	5	MYL7, ASPH, STIM1, SCN5A, TNNI3
	80µg	0,00292	5	MYH11, MYL7, TNNT2, ASPH, SCN5A
DNA repair	40 µg	0,0277	5	POLK, SUMO1, OGG1, BRCA2, EME2
